# Causal effect of renal function on venous thromboembolism: a two-sample Mendelian randomization investigation

**DOI:** 10.1007/s11239-021-02494-4

**Published:** 2021-05-27

**Authors:** Shuai Yuan, Maria Bruzelius, Susanna C. Larsson

**Affiliations:** 1 Unit of Cardiovascular and Nutritional Epidemiology, Institute of Environmental Medicine, Karolinska Institutet, Nobelsväg 13, 17177 Stockholm, Sweden; 2 Coagulation Unit, Department of Hematology, Karolinska University Hospital, Stockholm, Sweden; 3 Department of Medicine Solna, Karolinska Institutet, Stockholm, Sweden; 4 Department of Surgical Sciences, Uppsala University, Uppsala, Sweden

**Keywords:** Estimated glomerular filtration rate, Renal function, Venous thromboembolism, Mendelian randomization analysis

## Abstract

Whether renal function is causally associated with venous thromboembolism (VTE) is not yet fully elucidated. We conducted a two-sample Mendelian randomization (MR) study to determine the causal effect of renal function, measured as estimated glomerular filtration rate (eGFR), on VTE. Single-nucleotide polymorphisms associated with eGFR were selected as instrumental variables at the genome-wide significance level (*p* < 5 × 10^−8^) from a meta-analysis of 122 genome-wide association studies including up to 1,046,070 individuals. Summary-level data for VTE were obtained from the FinnGen consortium (6913 VTE cases and 169,986 non-cases) and UK Biobank study (4620 VTE cases and 356,574 non-cases). MR estimates were calculated using the random-effects inverse-variance weighted method and combined using fixed-effects meta-analysis. Genetically predicted decreased eGFR was significantly associated with an increased risk of VTE in both FinnGen and UK Biobank. For one-unit decrease in log-transformed eGFR, the odds ratios of VTE were 2.93 (95% confidence interval (CI) 1.25, 6.84) and 4.46 (95% CI 1.59, 12.5) when using data from FinnGen and UK Biobank, respectively. The combined odds ratio was 3.47 (95% CI 1.80, 6.68). Results were consistent in all sensitivity analyses and no horizontal pleiotropy was detected. This MR-study supported a casual role of impaired renal function in VTE.

**Supplementary Information:**

The online version contains supplementary material available at 10.1007/s11239-021-02494-4.

## Highlights

- Renal function has been associated with venous thromboembolism in observational studies.
- The present Mendelian randomization study confirmed a causal association between impaired renal function and elevated risk of venous thromboembolism in two independent populations.
- The findings highlight the importance of maintaining a healthy renal function in venous thromboembolism prevention.

## Introduction

Chronic kidney disease is one of the major global public health issues, affecting 9.1% (corresponding to 697.5 million) of individuals and causing 35.8 million disability-adjusted life years worldwide in 2017 [1]. Impaired renal function reflected by low estimated glomerular filtration rate (eGFR) is associated with changes in certain coagulant and inflammatory factors [2, 3] and has been revealed to be associated with an increased risk of venous thromboembolism (VTE) in most observational studies [4–7]. The association has been seen not only in individuals with chronic kidney disease [6, 7] but also in those with normal eGFR [4]. However, inherited methodological limitations, such as residual confounding and reverse causality, in observational studies challenge the causal inference on the effect of renal function measured by eGFR on risk of VTE.

Utilizing genetic variants as instruments for an exposure (e.g., eGFR), the Mendelian randomization (MR) framework can strengthen the causal inference in an exposure-outcome association by reducing residual confounding and diminishing reverse causality [8, 9]. The rationale for the reduction in unobserved confounding is that genetic variants are randomly allocated at conception, and therefore, one trait is generally not correlated with other traits. This process resembles the random assignment of participants to treatment and control groups in a randomized controlled trial [8, 9]. The MR design also minimizes reverse causality because alleles are fixed at birth and cannot be modified by the onset or progression of the disease [8, 9]. Therefore, if a genetic variant that alters the exposed level or imitates its biological effects is also associated with the disease (but not stronger than exposure), this offers strong evidence that the exposure is a causal risk factor for the disease. Here, we conducted a two-sample MR analysis to assess whether a decline in eGFR is causally associated with an elevated risk of VTE.

## Methods

### Study design

**Fig. 1:**
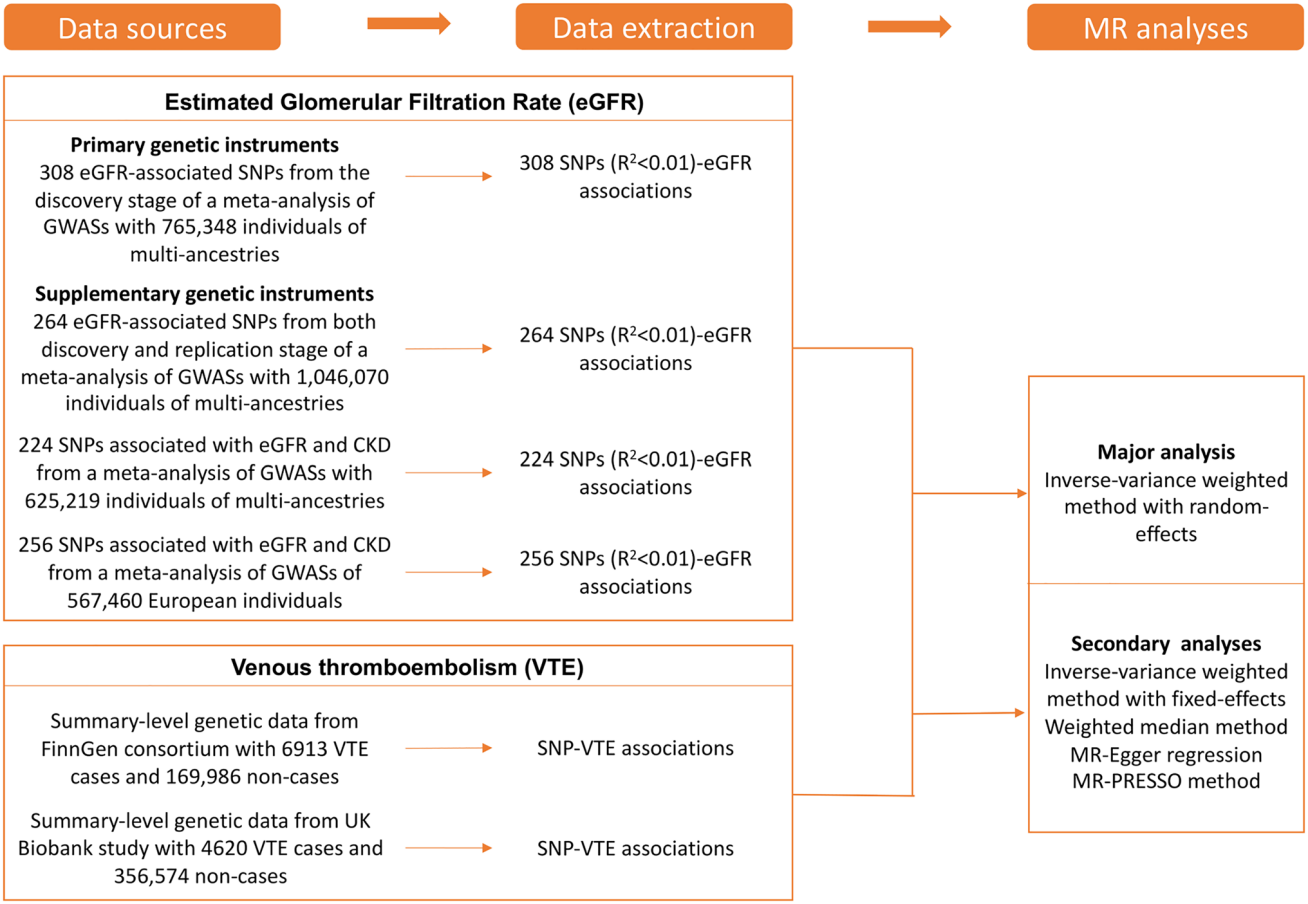
Study design overview. *eGFR* estimated glomerular filtration rate, *SNPs* single-nucleotide polymorphisms, *VTE* venous thromboembolism

The study design overview is shown in Fig. 1. Genetic instruments for eGFR were obtained from a meta-analysis of 122 genome-wide association studies (GWASs) with up to 1,046,070 individuals. Summary-level genetic data for VTE were derived from the FinnGen consortium and UK Biobank study. Detailed information on used studies or consortium are displayed in Supplementary table 1. Original studies included in the meta-analysis of GWASs for eGFR, the FinnGen consortium, and the UK Biobank study have been approved by a relevant review board. The present analyses were approved by the Swedish Ethical Review Authority.

### Genetic instrument selection

Single-nucleotide polymorphisms associated with creatinine-based eGFR were extracted as instrumental variables at the genome-wide significance level (*p* < 5 × 10^−8^) from the discovery trans-ancestry meta-analysis of 121 GWASs including up to 765,348 individuals of multi-ancestries (around 74% European population) and a replication study including up to 280,772 European-descent participants (> 90% men and many with comorbidities such as hypertension [10]) from the Million Veteran Program [11]. The 308 SNPs, explaining around 7.1% of phenotypic variance, identified in the discovery stage were proposed as the primary genetic instruments for eGFR. The median age of participants in the discovery stage was 54 years and a half of them were women. After the exclusion of participants with extreme eGFR values (< 15 and > 200 ml/min per 1.73 m^2^), the median of the study-specific mean eGFR values was 89 ml/min per 1.73 m^2^ and the interquartile range was 81–94 ml/min per 1.73 m^2^. Three supplementary sets of instruments, including 264 eGFR-associated SNPs from both discovery and replication stages, 224 replicated SNPs associated with both eGFR (*p* < 5 × 10^−8^) and chronic kidney disease (*p* < 0.05), and 256 eGFR-associated SNPs derived from European populations, were utilized to assess the robustness of the primary genetic instrument. Genome-wide association test adjusted for age, sex, study site, genetic principal components, relatedness and other study-specific features. All used SNPs were independent and not in linkage disequilibrium with a distance over 500-kb flanking regions and *r*^*2*^ ≤ 0.01 in the 1000Gp1v3 dataset. Detailed information on the four sets of genetic instruments for eGFR is presented in Supplementary table 2.

### VTE data sources

Summary genetic statistic data for VTE were available from the FinnGen consortium and UK Biobank study [12]. There was no participant overlap between the exposure and outcome populations. The FinnGen consortium (R4 release) includes 6913 VTE cases and 169,986 non-cases of Finnish descent. In FinnGen, individuals with ambiguous gender, high genotype missingness (> 5%), excess heterozygosity (± 4 standard deviation) and non-Finnish ancestry were excluded and the genetic variants with high missingness (> 2%), low Hardy–Weinberg equilibrium *p*-value (*p* < 5 × 10^−6^) and minor allele count, minor allele counts < 3 were excluded. Association tests were adjusted for age, sex, 10 genetic principal components and genotyping batch. Genotype imputation was done with the population specific SISu v3 reference panel. Detailed methods, including information on each included study, software used, fine-mapping and analytic codes, are presented in its website (https://www.finngen.fi/).

The data from the UK Biobank study were derived from Neale lab (the second wave, http://www.nealelab.is/uk-biobank) and encompasses 4620 VTE cases and 356,574 non-cases of British genetic ancestry. Individuals who are closely related (or at least one of a related pair of individuals) and those with sex chromosome aneuploidies were excluded. Neale lab restricted SNPs with minor allele frequency > 0.1% and Hardy–Weinberg equilibrium *p*-value > 1 × 10^−10^ and an info score > 0.8. The Haplotype Reference Consortium was used in imputation stage. There are around 54% female in all included participants. Association tests were adjusted for age, sex and up to 20 genetic principal components.

### Statistical analysis

The random-effects inverse-variance weighted method was used for the main analysis. This method provides estimate with the highest precision and rely on the assumption that all SNPs are valid instrumental variables [13]. Results from inverse-variance weighted random-effects model based on the FinnGen consortium and UK Biobank study were combined using fixed-effect meta-analysis method. Four other MR methods, including inverse-variance weighted fixed-effects model, weighted median method [14], MR-Egger regression [15] and MR-PRESSO method [16], were employed as sensitivity analyses to examine the robustness of the results and correct for pleiotropy. The weighted median approach can provide consistent estimates if ≥ 50% of the weight in the analysis comes from valid instrumental variables [14]. The MR-Egger regression can detect and correct for directional pleiotropy albeit with compromised power [15]. The MR-PRESSO test can detect possible outliers and generate estimates after outliers removing, thereby correcting for horizontal pleiotropy [16]. The MR-PRESSO distortion test aims at assessing the differences between the estimates before and after outlier correction and a *p* < 0.05 of distortion test indicates a significant difference in estimates before and after outlier correction [16]. Funnel and scatter plots were generated to visualize the directional pleiotropy [15]. The *I*^*2*^ (%) statistic [17] and Cochrane’s Q value was calculated to assess the heterogeneity among estimates across individual SNPs. Odds ratios (ORs) and corresponding confidence intervals (CIs) of VTE were scaled to one-unit decrease in log-transformed eGFR. All analyses were performed using the mrrobust package [18] in Stata/SE 15.0 (Stata Statistical Software: Release 15. College Station, TX: StataCorp LLC.) and the TwoSampleMR package [19] in R Software 3.6.0 (R Core Team. R Foundation for Statistical Computing. Vienna, Austria. 2019. https://www.R-project.org).

## Results

All eGFR-associated SNPs were available in the UK Biobank. Twenty-three SNPs were missing in the FinnGen consortium dataset of which twenty-two were replaced with proxy SNPs with *r*^*2*^ > 0.8. The proxy SNPs used are shown in Supplementary table 3. F-statistics for four sets of genetic instruments were over 10 (Supplementary table 2). Funnel plots showed a symmetric distribution of SNPs in both analyses based on the FinnGen consortium and UK Biobank study (Supplementary Fig. 1).

**Fig. 2:**
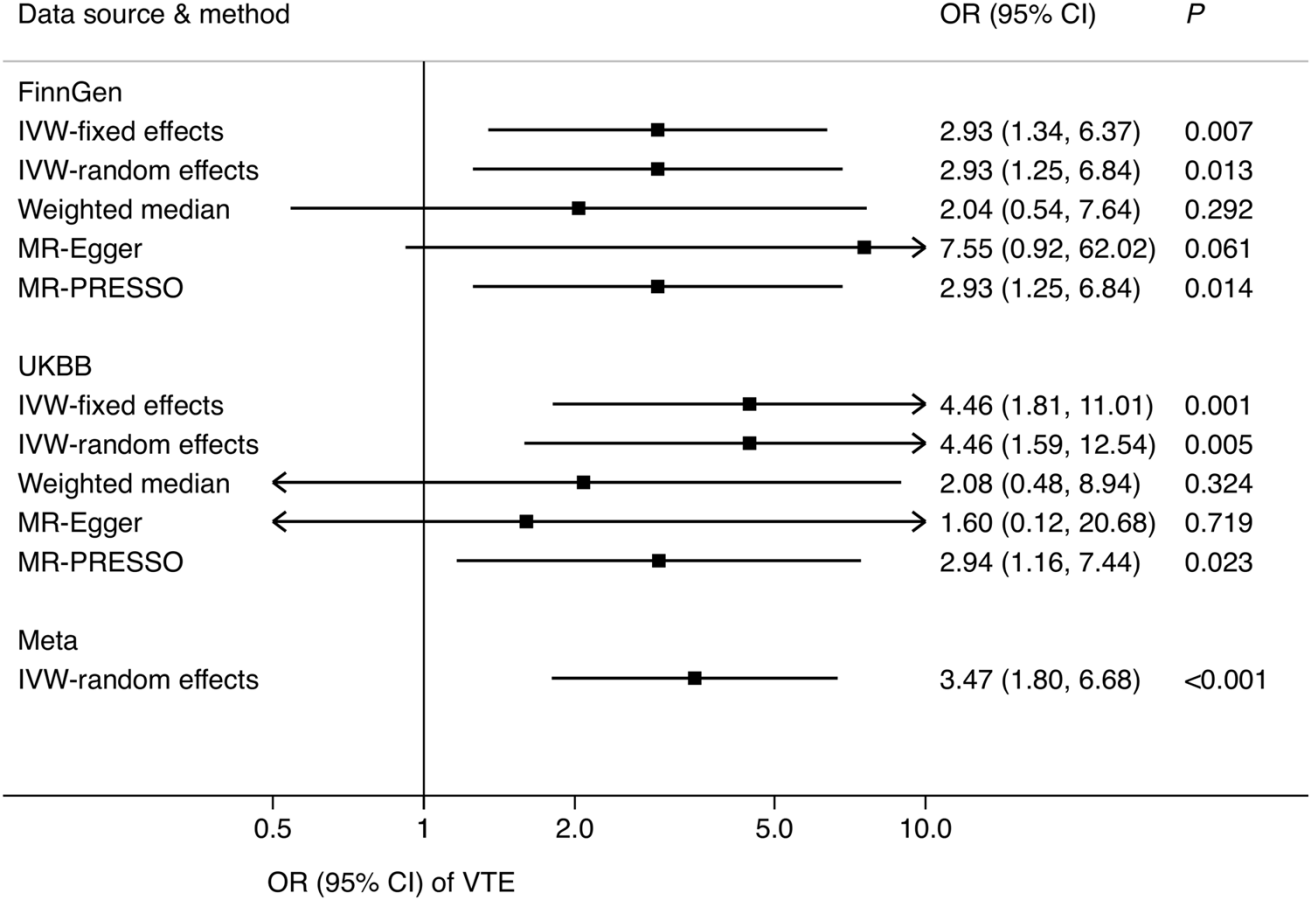
Association of genetically predicted eGFR with venous thromboembolism using 308 SNPs for eGFR. *CI* confidence interval, *eGFR* estimated glomerular filtration rate, *IVW* inverse-variance weighted, *OR* odds ratio, *SNPs* single-nucleotide polymorphisms. There were 10,023 venous thromboembolism cases and 486,809 non-cases in the meta-analysis of IVW-random effects model. Significant heterogeneity was detected among estimates from used SNPs in analyses based on data from both the FinnGen consortium (*I*^2^ = 19; Rucker’s Q = 377; *p*_het_ = 0.004) and UK Biobank study (*I*^*2*^ = 25; Rucker’s Q = 389; *p*_het_ < 0.001). There was no horizontal pleiotropy in MR-Egger regression using data from FinnGen consortium and UK Biobank study (*p* > 0.300). One and four outliers were detected in MR-PRESSO analyses using data from FinnGen consortium and UK Biobank study, respectively. However, *p* values for distortion tests were > 0.05, indicating no significant difference between estimates before and after outlier removing

Genetically predicted decreased eGFR was associated with an increased risk of VTE in both the FinnGen consortium and UK Biobank (Fig. 2). For one-unit increase in log-transformed eGFR, the ORs of VTE were 2.93 (95% CI 1.25, 6.84; *p* = 0.013) using data from FinnGen and 4.46 (95% CI 1.59, 12.5; *p* = 0.005) using data from UK Biobank. The combined OR was 3.47 (95% CI 1.80, 6.68; *p* < 0.001) in the meta-analysis. Results were consistent in all sensitivity analyses. Significant heterogeneity was detected across estimates from used SNPs in analyses based on data from both FinnGen (*I*^2^ = 16; Cochrane’s Q = 377; *p*_het_ = 0.004) and UK Biobank (*I*^2^ = 25; Cochrane’s Q = 389; *p*_het_ < 0.001). There was no horizontal pleiotropy in MR-Egger regression (*p* > 0.300). In the MR-PRESSO analyses, one and four outliers were detected using data from FinnGen and UK Biobank, respectively. However, *p* values for distortion tests were > 0.05, indicating no significant difference between estimates before and after outlier removal. Scatter plot of the association of eGFR with VTE using 308 SNPs is shown in Supplementary Fig. 2.

Results of analyses using three supplementary sets of genetic instrumental variables for eGFR are presented in Table 1. Overall, the associations of genetically predicted eGFR with VTE risk persisted in all analyses based on the inverse-variance weighted method albeit with significant heterogeneity among used SNPs (*p* < 0.05). We did not detect horizontal pleiotropy in all MR-Egger regression (*p* > 0.05). One to six outliers were observed in MR-PRESSO analyses. However, all *p* values for distortion tests were > 0.05.

**Table 1:** Associations between genetically predicated eGFR and venous thromboembolism using supplementary genetic instruments for eGFR

Data source	Genetic instruments	Method	OR	95% CI	*P*
FinnGen consortium	264 SNPs from both discovery and replication stages	IVW-fixed model	3.41	1.52, 7.67	0.003
		IVW-random model	3.41	1.44, 8.10	0.005
		Weighted median	1.95	0.52, 7.33	0.322
		MR-Egger	4.11	0.49, 34.4	0.193
		MR-PRESSO	3.41	1.44, 8.10	0.006
	224 SNPs associated with both eGFR (*p* < 5 × 10^−8^) and chronic kidney disease (*p* < 0.05)	IVW-fixed model	2.94	1.25, 6.90	0.013
		IVW-random model	2.94	1.15, 7.53	0.024
		Weighted median	1.81	0.44, 7.50	0.411
		MR-Egger	3.39	0.35, 32.7	0.293
		MR-PRESSO	2.94	1.15, 7.53	0.025
	256 SNPs identified from European population	IVW-fixed model	2.52	1.16, 5.48	0.020
		IVW-random model	2.52	1.05, 6.02	0.038
		Weighted median	1.65	0.48, 5.64	0.425
		MR-Egger	1.56	0.17, 14.2	0.696
		MR-PRESSO	2.29	0.97, 5.40	0.059
UK Biobank study	264 SNPs from both discovery and replication stages	IVW-fixed model	3.85	1.52, 10.0	0.005
		IVW-random model	3.85	1.33, 11.1	0.013
		Weighted median	1.92	0.41, 9.09	0.403
		MR-Egger	1.49	0.11, 20.0	0.766
		MR-PRESSO	2.22	0.88, 5.56	0.093
	224 SNPs associated with both eGFR (*p* < 5 × 10^−8^) and chronic kidney disease (*p* < 0.05)	IVW-fixed model	4.00	1.49, 11.1	0.006
		IVW-random model	4.00	1.27, 12.5	0.018
		Weighted median	1.47	0.3, 7.14	0.636
		MR-Egger	2.13	0.13, 33.3	0.593
		MR-PRESSO	2.27	0.85, 5.88	0.105
	256 SNPs identified from European population	IVW-fixed model	4.17	1.72, 10.0	0.002
		IVW-random model	4.17	1.45, 12.5	0.008
		Weighted median	2.13	0.52, 9.09	0.291
		MR-Egger	1.27	0.08, 20.0	0.867
		MR-PRESSO	2.17	0.90, 5.26	0.084

*CI* confidence interval, *eGFR* estimated glomerular filtration rate, *IVW* inverse-variance weighted, *OR* odds ratio, *SNPs* single-nucleotide polymorphisms. We detected significant heterogeneity among used SNPs in all inverse-variance weighted models (*p* < 0.05), but no horizontal pleiotropy in all MR-Egger regression (*p* > 0.05). One to six outliers were detected in MR-PRESSO analyses. However, all *p* values for distortion tests were > 0.05, indicating no significant difference between estimates before and after outlier removing

## Discussion

**Fig. 3:**
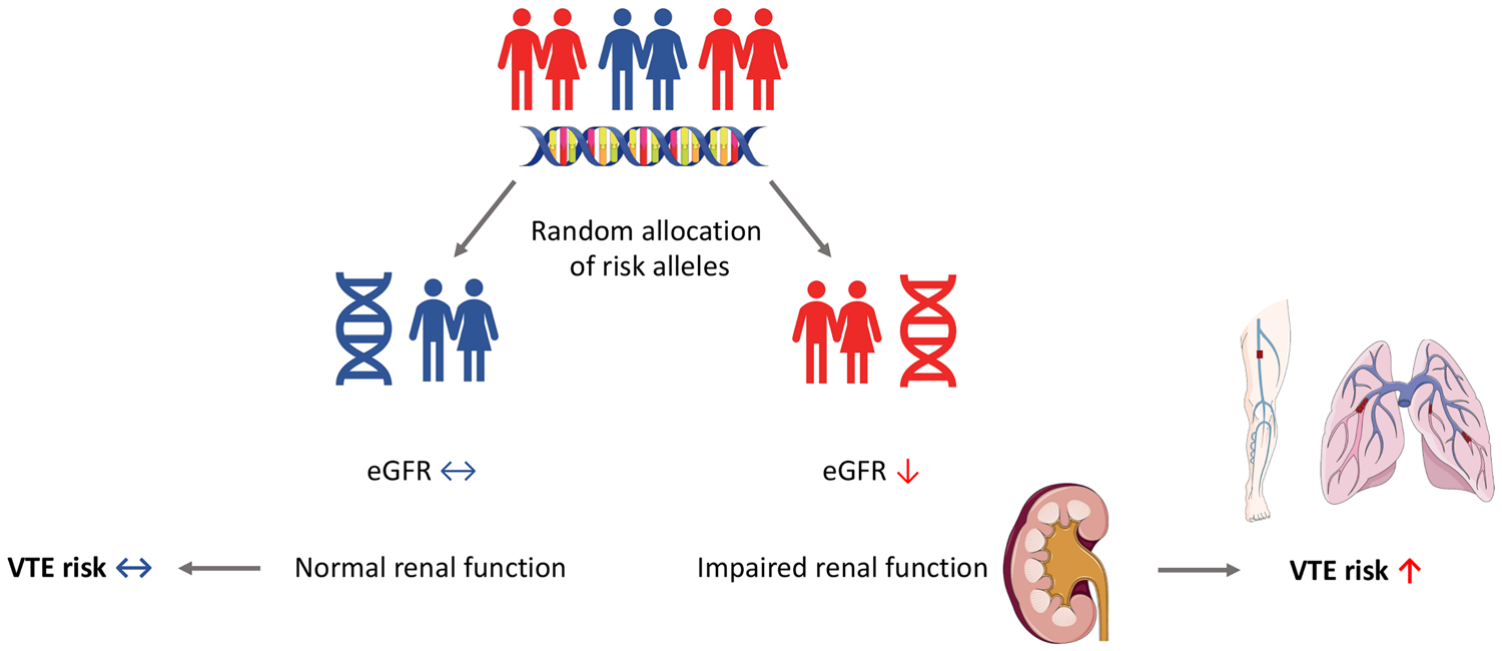
Summarizing figure on MR association between eGFR and VTE risk. *eGFR* estimated glomerular filtration rate, *MR* Mendelian randomization, *VTE* venous thromboembolism

The present two-sample MR study strengthened the evidence of a causal association between eGFR and VTE (Figs. 2, 3). The association was consistent in two independent populations and remained stable using different sets of instrumental variables for eGFR and in all statistical models.

Our finding is in line with observational studies [4–7]. The Longitudinal Investigation of Thromboembolism Etiology study including 19,073 middle-aged and elderly adults found that individuals with stage 3/4 chronic kidney disease (eGFR between 15 and 59 ml/min per 1.73 m^2^) had 28% higher risk of VTE compared with those with normal renal function in a follow-up period of 11.8-year [7]. A large pooled individual participant data additionally revealed a dose–response relationship between eGFR and VTE risk and specified that eGFR could be an independent predictor for VTE risk even among individual with normal eGFR range [4]. The present study used MR design empowered the causal inference in the association between eGFR and VTE and confirmed the protective effect of high eGFR on VTE.

There are several potential biological pathways underlying the link between eGFR and VTE [2, 20]. In particular, pre-coagulation is activated in patients with insufficient renal function, and this may accelerate the development of VTE. Impaired kidney function is associated with elevated levels of coagulation factors, such as D-dimer, fibrinogen, factor VII, and factor VIII and von Willebrand factor [2, 3, 21, 22]. On the other hand, endogenous anticoagulants, such as antithrombin, are decreased among individuals with impaired kidney function due to increased urinary loss of antithrombin out of proportion to synthesis [23]. Change in antithrombin, however, was not observed in one case–control study [3] and the alternation of anticoagulants might occur in severe chronic kidney disease mainly. Hypoalbuminemia caused by kidney disease (nephrotic syndrome) results in increased availability of thromboxane A2 that enhances platelet activation and aggregation, thereby facilitating thrombus formation [24]. In addition, some studies indicated that decreased activity of the fibrinolytic system might also mediate the association between renal function and VTE. However, data on this pathway are inconclusive and needs to be unified [25, 26].

There are several strengths and limitations of the present study. The major merit is the MR design which greatly strengthened the causal inference on the associations between eGFR and VTE by reducing residual confounding and reverse causality. Additionally, the association was tested and replicated in two independent data sources. The high consistency between findings from FinnGen consortium and UK Biobank study gave a boost to the possibility that the finding is causal. A limitation is that there might be population bias in the finding using genetic instruments derived from population of multi-ancestries. However, a high proportion of European population (around 74%) and a consistent result based on merely European population minimized the possibility that our finding was affected by population bias. The GWAS meta-analysis on eGFR found that eGFR was genetically correlated with lean mass and physical fitness (*r*_*g*_ =  − 0.20), which might be a pleiotropic factor influencing the established association between eGFR and VTE. However, a null association between lean mass and VTE in a recent MR study [27] and no directional pleiotropy detected in the present MR-Egger regression analysis indicated a negligible distortion by potential pleiotropy. Chronic kidney disease and VTE are both frequently observed in cancer patients [28, 29]. We cannot rule out that the eGFR-associated SNPs have pleiotropic associations with cancer, and that the observed association between eGFR and VTE is to some extent influenced by cancer. In addition, as we used summary-level data, we were unable to assess the dose–response relationship between eGFR and VTE and could not stratify the analyses by impaired renal function.

## Conclusions

The present study provides the first MR evidence that declined renal function, measured as decreased eGFR, is causally associated with an increased risk of VTE. The finding suggests that clinicians need to optimize medical treatment to maintain healthy renal function of patients to prevent future VTE and VTE screening and prophylaxis should be reinforced among individuals with impaired renal function.

## Supplementary Information

Below is the link to the electronic supplementary material.

Supplementary file1 (DOCX 703 kb)

## Data Availability

The datasets analyzed in this study are publicly available summary statistics. Data used in the present study are available in OSF data respiratory (https://osf.io/bk52p/).
