# Supplementary material for: Causal effect of renal function on venous thromboembolism: a two-sample Mendelian randomization investigation
Source: J Thromb Thrombolysis. 2021 May 27;53(1):43–50. doi: 10.1007/s11239-021-02494-4 (PMC8791872; doi:10.1007/s11239-021-02494-4)
Supplement: Supplementary file 1 — Supplementary file1 (DOCX 703 kb) [file 11239_2021_2494_MOESM1_ESM.docx]

**Supplements**

**Causal effect of renal function on venous thromboembolism: a two-sample Mendelian randomization investigation**

*Shuai Yuan, Maria Bruzelius, Susanna C. Larsson*

**Supplementary Table 1.** Detailed information on used studies in the present study

**Supplementary Table 2**. Different sets of genetic instruments selected for eGFR

**Supplementary Table 3.** Proxy SNPs used in the present study

**Supplementary Figure 1.** Funnel plot of the association between genetically predicted eGFR and venous thromboembolism using 308 SNPs for eGFR

**Supplementary Figure 2.** Scatter plot of the association between genetically predicted eGFR and venous thromboembolism using 308 SNPs for eGFR

**Supplementary Table 1.** Detailed information on data sources used in the present study

| **Exposure/Outcome** | **Consortium or cohort study** | **Participants** | **PubMed ID or**  **web source** |
| --- | --- | --- | --- |
| Genetic instruments for eGFR | Meta-analysis of 121 genome-wide association studies in the discovery stage and one replication study | Detailed information is provided in Supplementary table 1 | PubMed ID: [31152163](https://www.nature.com/articles/s41588-019-0407-x#Sec2) |
| Summary-level data for venous thromboembolism | FinnGen consortium | 6913 venous thromboembolism cases and 169,986 non-cases of Finnish ancestry | <https://www.finngen.fi/fi> |
|  | UK Biobank study | 4620 venous thromboembolism cases and 356,574 non-cases of European ancestry | <https://www.ukbiobank.ac.uk/>  Data obtained from Neale lab http://www.nealelab.is/ |

eGFR indicates estimated glomerular filtration rate**;** PubMed ID, PubMed identifier.

**Supplementary Table 2.** Different sets of genetic instruments selected for eGFR

| **Different sets of genetic instruments for eGFR** | **Number of participants included in genotyping** | **Total SNPs** | **Used SNPs in FinnGen consortium** | **Used SNPs in UK Biobank study** | **Overall**  **F-statistic** |
| --- | --- | --- | --- | --- | --- |
| 308 SNPs from the discovery stage | 765,348 | 308 | 307 | 308 | 76.2 |
| 264 SNPs from both discovery and replication stages | 1,046,070 | 264 | 263 | 264 | 81.3 |
| 224 SNPs associated with both eGFR (*p*<5×10^-8^) and chronic kidney disease (*p*<0.05) | 625,219 | 224 | 223 | 224 | 86.3 |
| 256 SNPs identified from European population | 567,460 | 256 | 255 | 256 | 60.7 |

eGFR indicates estimated glomerular filtration rate; SNPs, single-nucleotide polymorphisms. We estimated F-statistic using following formula: F-statistic = $\frac{(N-k-1)}{K}$ * $\frac{R^2}{1-R^2}$ (N means sample size; k, number of SNPs; R^2^, phenotypic variance explained by used SNPs).

**Supplementary Table 3.** Proxy SNPs used in the present study

| **Original SNP** | **Proxy SNP** | **R^2^** | **Chromosome** |
| --- | --- | --- | --- |
| rs34720381 | rs35039375 | 1.00 | 1 |
| rs78614739 | rs34502618 | 1.00 | 1 |
| rs6722113 | rs7586645 | 1.00 | 2 |
| rs13029395 | rs35483597 | 0.94 | 2 |
| rs9838792 | rs7647657 | 1.00 | 3 |
| rs11914389 | rs6599206 | 1.00 | 3 |
| rs3750081 | rs10260000 | 1.00 | 7 |
| rs801193 | rs4636083 | 1.00 | 7 |
| rs60991551 | rs72650425 | 1.00 | 8 |
| rs13287061 | rs9657704 | 1.00 | 9 |
| rs12240572 | rs59958877 | 1.00 | 10 |
| rs11237450 | NA | Not biallelic in Europeans | 11 |
| rs11063193 | rs1029769 | 1.00 | 12 |
| rs10850001 | rs17630235 | 0.88 | 12 |
| rs303937 | rs303938 | 1.00 | 13 |
| rs28522606 | rs28626665 | 1.00 | 15 |
| rs4886696 | rs3809547 | 1.00 | 15 |
| rs193538 | rs246234 | 1.00 | 16 |
| rs28735420 | rs6502182 | 1.00 | 17 |
| rs2411192 | rs34675748 | 1.00 | 17 |
| rs9907229 | rs7214227 | 1.00 | 17 |
| rs62187541 | rs56256914 | 1.00 | 20 |
| rs131263 | rs131289 | 1.00 | 22 |

SNP indicates single-nucleotide polymorphism. Proxy SNPs were searched in the database of Division of Cancer Epidemiology & Genetic, National Cancer Institute (<https://ldlink.nci.nih.gov/?tab=home>). We set R^2^ >0.80 as the threshold for suitable proxy SNP.

**Supplementary Figure 1.** Funnel plot of the association between genetically predicted eGFR and venous thromboembolism using 308 SNPs for eGFR

**
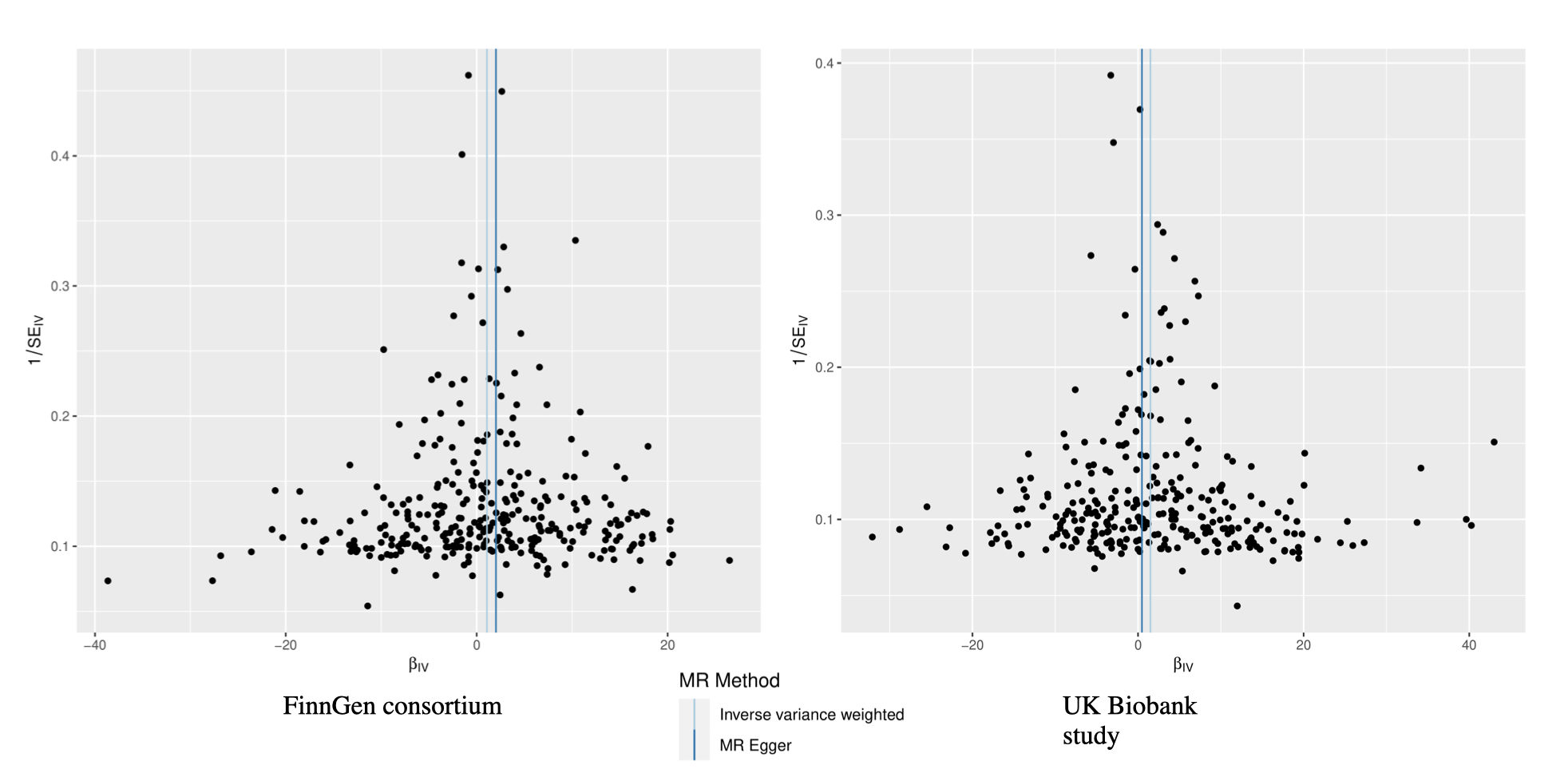
**

eGFR indicates estimated glomerular filtration rate; SE, standard error; SNPs, single-nucleotide polymorphisms.

**Supplementary Figure 2.** Scatter plot of the association between genetically predicted eGFR and venous thromboembolism using 308 SNPs for eGFR

**
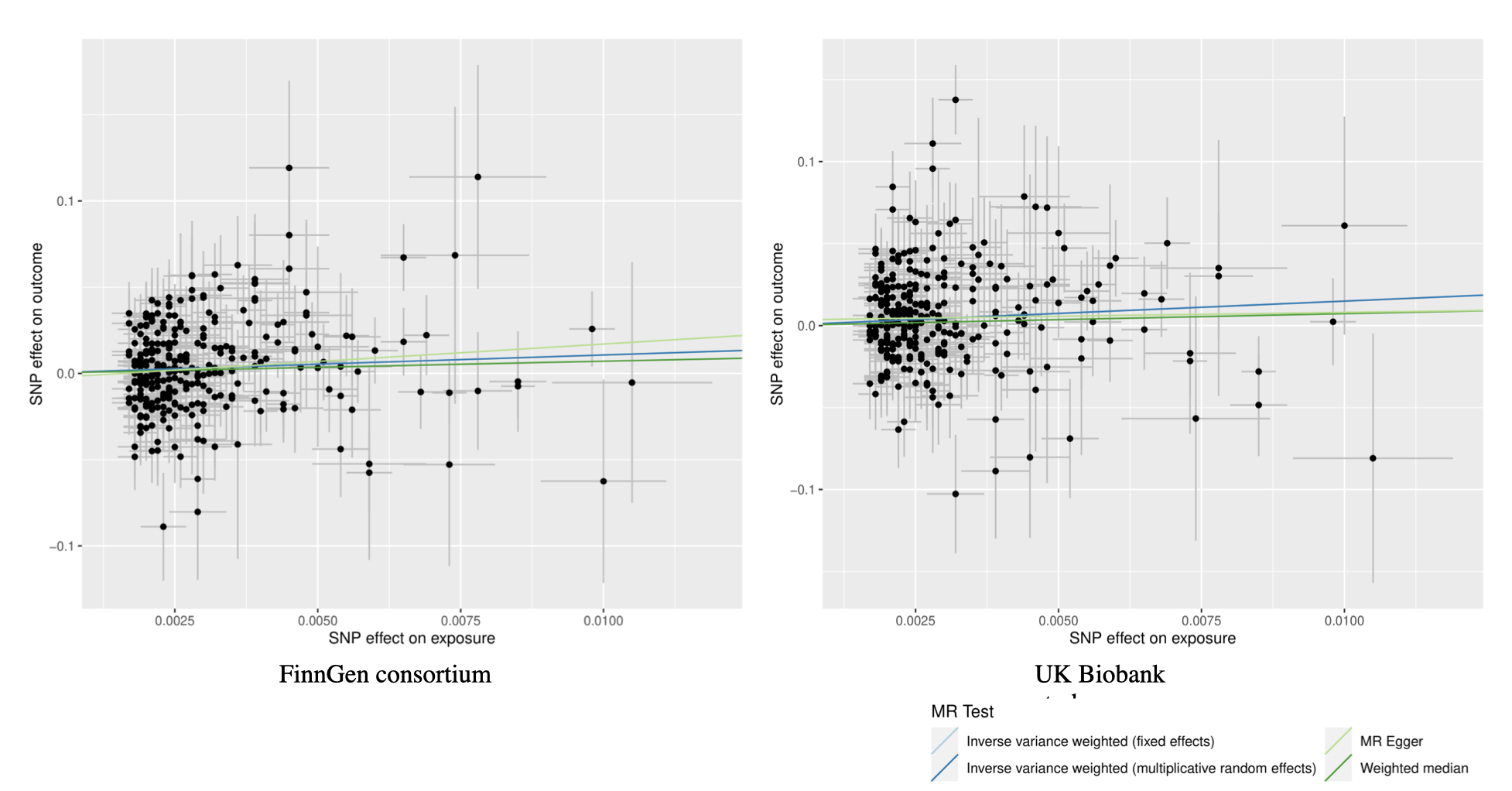
**

eGFR indicates estimated glomerular filtration rate; SNPs, single-nucleotide polymorphisms.
